# Comparative genomic profiling of *CBFs* pan-gene family in five yellowhorn cultivars and functional identification of *Xg11_CBF11*

**DOI:** 10.3389/fpls.2024.1481358

**Published:** 2024-11-19

**Authors:** Juan Wang, Xizhen Liang, Weiyang Zhang, Asma Khalil, Yingying Wu, Sisi Liu, Muhammad Tahir ul Qamar, Xingqiang Wang, Jinping Guo

**Affiliations:** ^1^ College of Forestry, Shanxi Agricultural University, Taigu, Shanxi, China; ^2^ Integrative Omics and Molecular Modeling Laboratory, Department of Bioinformatics and Biotechnology, Government College University Faisalabad (GCUF), Faisalabad, Pakistan

**Keywords:** *Xanthoceras sorbifolium*, pangenome-wide, gene ontology, low-temperature stress, physiological response, transgene

## Abstract

C-repeat binding factor (*CBF*) transcription factors can activate the expression of a series of cold regulation-related genes, thereby improving the cold resistance of plants. However, no detailed information is known about the biological functions of CBF proteins in yellowhorn (*Xanthoceras sorbifolium*). In this study, a total of 59 *CBF* gene family members were identified in five yellowhorn cultivars *(WF18, Zhongshi 4, Jinguanxipei 2021, Zhong Guan NO.2*, and *XsoG11)*, revealing their intraspecific structural and functional diversity, with 8 core genes present in all cultivars. Phylogenetic and motif analyses highlighted conserved features and species-specific adaptations. Gene duplication events revealed that tandem duplicates are major factors involved in the expansion of this gene family in yellowhorn. Expression profiling under stress conditions demonstrated the involvement of these genes in stress responses. Of particular interest was *Xg11_CBF11*, which showed strong induction by low-temperature stress. Overexpression of *Xg11_CBF11* in *Arabidopsis thaliana* was performed to validate its cold resistance function. The wild-type and T2 transgenic *A. thaliana* plants were subjected to low-temperature stress at 4°C for 0, 24, and 48 h, and physiological indexes related to antioxidant enzyme activity, photosynthesis, and cell membrane permeability were determined by comparative test. The results were as follows: the POD and SOD activities of transgenic lines were significantly higher than those of wild-type lines, indicating *Xg11_CBF11* improved the adaptability of *A. thaliana* to low-temperature; The increase of relative conductivity and malondialdehyde, the decrease of chlorophyll content in transgenic lines were smaller than those of wild-type lines, indicating *Xg11_CBF11* enhanced the resistance of *A. thaliana* to low-temperature stress. These results implied that *Xg11_CBF11* has a positive regulatory effect on *A. thaliana* ‘s response to low-temperature stress.

## 1 Introduction

AP2/ERF is a widely known gene family that regulates responses to environmental stresses in higher plants. Several reports have shown that plants with these overexpressed factors show better tolerance to environmental stresses (Feng et al., 2020). Over time it has evolved into a signal transduction pathway, the ICE1-CBF-COR pathway. When plants are subjected to abiotic stress such as cold, the ICE1, which is the inducer of CBF expression binds to the MYC recognition site. The ICE1 encodes MYC-like bHLH transcriptional activator which binds to the sequence in MYC. This MYC recognition sequence is present in the promoter sequence of the *CBF* genes and helps activate transcription. The overexpression of CBF proteins activates the COR (cold-responsive) genes and helps plants tolerate cold stress (Chinnusamy et al., 2010; Verma et al., 2020).

The *AP2/ERF* genes contain AP2/ERF DNA binding domains which are 60-70 amino acids long. These domains contain RAYD and YRG motifs. Based on similarity among amino acid sequences and the domains, the *AP2/ERF* genes can be classified into four categories. The sequences belonging to AP2 family contain two AP2 domains. The members of EREBP family contain one AP2 domain (Kagaya et al., 1999). The EREBP family is further subdivided into DREB and ERF subfamilies. The RAV family members contain B3 and AP2 domains. These RAV family members are regulated by brassinosteroid or ethylene and regulate responses to environmental stresses (Hu et al., 2022). The Soloist family members contain single AP2 domain and participate in disease regulatory mechanisms (Giri et al., 2014). Thus, these TFs are involved in various defense-related mechanisms and induce responses to various stimuli including salt, drought, and heat stress. The *CBF* genes contain two AP2 domains which contain the transcriptional activator. This transcriptional activator binds to the CCGAC, the low-temperature-responsive element, thereby, inducing the expression of some genes related to cold stress responsiveness (Lv et al., 2020).

In *A. thaliana*, three *CBF* genes; *CBF1*, *CBF2*, and *CBF3* have been reported to show cold tolerance (Lv et al., 2020). Other plant species that exhibit cold tolerance contain signature sequences in CBF-like proteins. CBF orthologs express rapidly in *Brassica napus* (Gao et al., 2002), barley (J. Ding et al., 2021), tomato (Pontaroli et al., 2009), and rice (Dubouzet et al., 2003). An *O. sativa* AP2/ERF gene, *OsDREBL* was cloned and showed responsiveness to cold stress (Chen et al., 2003). In wild soybean, the member of ERF family is involved in alkaline stress tolerance. The *GsERF6* gene was introduced in *A. thaliana* and induced tolerance to alkaline stress (Yu et al., 2016).

Yellowhorn, belonging to the genus *Xanthoceras*, is a deciduous shrub or small tree, as well as a unique woody oil tree in China. The oil content of the seed kernel can reach about 60%, which can be used as high-quality raw material for advanced edible oil and biodiesel, and has high economic use value (Li et al., 2022). As an important oil tree species, the main research content focuses on oil metabolism, processing and extraction, and seedling cultivation. Cold stress influences the plant’s survival by causing cellular dehydration in tissues. Plants can respond to cold stress through a wide range of transcriptional changes to maintain cellular homeostasis and improve cold tolerance. Still, there are few studies on the internal mechanism of cold resistance in yellowhorn. The cold resistance function of *CBF* transcription factor genes has been demonstrated in various plants. Under freezing or chilling temperature conditions, *CBFs* induce several cold-stress-responsive genes that provide tolerance against this stress. However, the roles of CBFs in yellowhorn still remains unclear.

The identification studies of yellowhorn *CBF* genes are important as *CBF* genes play vital roles in stress resistance and have not been identified in yellowhorn. Traditional approaches for gene family identification studies utilize a single reference genome and, thus, cannot identify the members of gene families present in other genomes but absent in the reference genome (Ding et al., 2024). Wang et al. reported the assembly as well as comparative pan-genome-wide gene family studies among yellowhorn and other species genomes, providing an important resource for further functional studies (Wang et al., 2023, 2024; Hu et al., 2024; Jia et al., 2023).

The current study reports the pan-genome-wide identification of *CBF* genes from five yellowhorn cultivars including *WF18 (Xwf8), Zhongshi 4 (Xzs4), Jinguanxipei 2021 (Xjg), Zhoug Guan NO.2 (Xzg2)*, and *XsoG11 (Xg11)*. These genes were further investigated for intraspecies diversity and functional conservation. Further, the expression level of these genes was checked in seed tissues and under abiotic stress. We focused on *Xg11_CBF11*, which showed strong induction under low-temperature stress. Based on the cloning of the *Xg11_CBF11* gene, expression vector was constructed and was transformed into *A. thaliana*. The transgenic plants were subjected to cold stress to verify the cold resistance function of the *Xg11_CBF11* gene, by comparing the changes in antioxidant enzyme activity, chlorophyll content, relative conductivity, and malondialdehyde content. The analysis of the physiological indicators of cold resistance in *Xg11_CBF11*-overexpressing *A. thaliana* is the expansion and deepening of the molecular mechanism of cold resistance in yellowhorn.

## 2 Materials and methods

### 2.1 Pan-genome-wide identification of *CBF* gene family members in five yellowhorn cultivars and analysis of their physicochemical properties

Protein sequences of CBF from *A. thaliana, O. sativa*, and *Triticum aestivum* were obtained from the NCBI Protein database (https://www.ncbi.nlm.nih.gov/protein/) (Geer et al., 2010). These sequences were used as queries for a local BLASTp search (Altschul et al., 1997) against five yellowhorn cultivars (*Xzs4, Xwf8, Xjg, Xg11*, and *Xzg2*). To confirm the presence of the full AP2 domain, the candidate sequences were analyzed using the Pfam (https://pfam.xfam.org/) (Mistry et al., 2021), CDD (https://www.ncbi.nlm.nih.gov/Structure/cdd/wrpsb.cgi) (Marchler-Bauer et al., 2015), and SMART (http://smart.embl-heidelberg.de/) (Schultz et al., 2000) databases. Sequences that contained the complete AP2 domain were finalized for subsequent analysis and named according to their chromosomal order. The physicochemical properties of the CBF proteins were predicted using the ExPASy ProtParam tool (https://web.expasy.org/protparam/) (Gasteiger et al., 2005). Subcellular localization predictions were made using the WoLF PSORT tool (https://wolfpsort.hgc.jp/) (Horton et al., 2007).

### 2.2 Phylogenetic analysis of yellowhorn *CBFs*

Using the default settings of ClustalW, CBF protein sequences from *A. thaliana, O. sativa, Lolium perenne, Hordeum vulgare, T. aestivum, Secale cereale*, and five yellowhorn cultivars were aligned (Kumar et al., 2016; Zameer et al., 2022). A phylogenetic tree was then constructed using the Maximum Likelihood (ML) method with 1000 bootstrap replicates in the IQ-TREE Web Server, and JTT+F+I+G4 was selected as the best-fit substitution model based on the BIC scores (http://iqtree.cibiv.univie.ac.at/) (Trifinopoulos et al., 2016). The resulting phylogenetic tree was edited and visualized using iTOL: Interactive Tree of Life v6 (https://itol.embl.de/) (Sadaqat et al., 2024; Letunic and Bork, 2021).

### 2.3 Exon-Intron representation and assessment of conserved motif

The gene structures of *CBFs* were analyzed using the Gene Structure View (advanced) tool in Tbtools, with the annotation file as input (Chen et al., 2020). Conserved motifs of CBFs were identified using the amino acid sequences as input in the MEME website (https://meme-suite.org/) (Bailey et al., 2015), with the maximum number of motifs set to 20.

### 2.4 Chromosomal localization, Ka/Ks ratios, gene duplication analysis

The chromosomal positions of all *CBF* genes in the five yellowhorn cultivars were obtained from their annotation files. The positional localization of the *CBF* genes was performed and visualized using TBtools software (Chen et al., 2018). Gene duplication events for *CBF* genes were identified based on the criteria that the shorter gene covered at least 70% of the length of the longer gene and that the similarity between the two aligned genes was at least 70% (Tahir and Sadaqat, 2023). Tandem and segmental duplications were reported as the two main mechanisms underlying gene family expansion. The synonymous substitution rate (Ks), nonsynonymous substitution rate (Ka), and the Ka/Ks ratio were used to assess the selection history and duplication events (Fatima et al., 2023). The Ks and Ka values of duplicated *CBF* genes were computed using DnaSP v6 (Rozas et al., 2017). The following formula was used to calculate the duplication time: T= Ks/(2 × 1.5 × 10^−8^)*10^−6^ million years ago (Mya) (Ma et al., 2024; Yang et al., 2024).

### 2.5 Protein-Protein interaction and gene ontology enrichment analysis

The amino acid sequences of the yellowhorn *Xg11* variety were used to analyze the interactions among CBF proteins using STRING database (Mering et al., 2003). The top 10 interactions were kept and 0.4 was selected as the interaction threshold. Cytoscape software (Shannon et al., 2003) was used to visualize the interactions. The components of GO enrichment were predicted using PANNZER database (Törönen et al., 2018).

### 2.6 Prediction of *cis*-regulatory elements and expression profiling of *Xg11 CBFs*

The 2kb upstream regions of yellowhorn *CBFs* genes were retrieved and searched for *cis*-elements using the PlantCARE database (http://bioinformatics.psb.ugent.be/webtools/plantcare/html/) (Rombauts et al., 1999; Zia et al., 2024). The RNA-seq data of yellowhorn *Xg11* cultivar was analyzed to check the expression profiles of *CBF* genes in low and high-temperature stress; drought stress (BioProject: PRJNA974867); salt and alkali stress; and tissues (BioProject: PRJNA923394). The transcriptomic data was obtained from SRA-NCBI database (https://www.ncbi.nlm.nih.gov/sra) and the quality of reads was evaluated using FastQC tool (Wingett and Andrews, 2018). The genome was indexed and the clean-reads were mapped onto it using HISAT (Kim et al., 2019). StringTie (Pertea et al., 2016) was further used to estimate the expression in fragments per kilobase of transcript per million mapped reads (FPKM) values (Sadaqat et al., 2023).

### 2.7 Plant materials and treatments

The study utilized yellowhorn seedlings and wild-type *A. thaliana* plants. The *Xg11_CBF11* gene was introduced into *A. thaliana*, resulting in T2 transgenic lines (L1, L5, L6) with high expression. “G11” (superior line of yellowhorn) seeds were collected from Shanxi Agricultural University, sown, and grown until seedlings reached about 15 cm. Leaves of yellowhorn seedlings treated at 4°C for 12 hours were harvested for cloning *Xg11_CBF11*. Wild-type *A. thaliana* seeds were grown in a light incubator with a relative humidity of 60%-70%, a photoperiod of 16/8h, and a temperature of 25°C. Both wild-type and transgenic *A. thaliana* lines were treated with 4°C cold stress and sampled at 0, 24, and 48h. Various physiological indicators, including antioxidant enzyme activity (POD, SOD), chlorophyll content, relative conductivity, and malondialdehyde content, were measured.

### 2.8 Gene cloning and transformation

For gene cloning and transformation, RNA was extracted from yellowhorn seedlings subjected to cold stress using the RNAprep pure Plant Kit (Tiangen, Beijing, China). cDNA was synthesized using the Fastking reverse transcription kit, TaKaRa, Dalian, China, and the *Xg11_CBF11* gene was amplified using specific primers designed from the full-length CDS sequence. The specific primer pairs (*Xg11_CBF11*-F/R) of the selected genes were designed using Primer 3.0 software (https://bioinfo.ut.ee/primer3-0.4.0/) (**Supplementary Table S1**) and synthesized by Tsingke Biotech. The amplified product was inserted into the pMD19-T vector and transformed into *E. coli* DH5α for cloning.

The *Xg11_CBF11* gene was then inserted into the pEGOEP35S-H vector with *Xg11_CBF11*-e-F/R primers (**Supplementary Table S1**), and *Agrobacterium tumefaciens* containing the recombinant plasmid was used to infect *A. thaliana* plants through the floral-dip method. Transgenic seeds (T1) were selected on a hygromycin-containing medium and further confirmed by PCR (Ding et al., 2021, 2023).

To precisely quantify the expression levels of *Xg11_CBF11* in transgenic *A. thaliana*, a quantitative real-time polymerase chain reaction (qRT-PCR) was conducted employing specifically designed *Xg11_CBF11*-q-F/R primers (**Supplementary Table S1**). For normalization and accurate quantification, *AtActin2*, a constitutively expressed gene from *A. thaliana*, was utilized as the internal reference gene.

### 2.9 Determination of physiological indicators and data analysis

To determine physiological indicators, fresh leaves (0.2 g) were ground, and enzyme activities were measured using specific kits (Solarbio, Beijing, China). Absorbance values were recorded at 470 nm and 560 nm for POD and SOD, respectively. Chloroplast pigments were extracted with 95% ethanol, and absorbance values were measured at 665 nm and 649 nm to calculate chlorophyll a and chlorophyll b content. Leaf strips were soaked in deionized water for 12 h, and conductivity was measured before and after boiling to assess cell membrane permeability. Malondialdehyde (MDA) was determined using thiobarbituric acid, with absorbance measured to calculate content, indicating lipid peroxidation levels. The physiological data were analyzed using ANOVA in SPSS software. Mean and standard error values were plotted using Origin software to visualize the trends and differences between treatments.

## 3 Results

### 3.1 Pan-genome−wide Identification of *CBF* genes from five yellowhorn cultivars

A total of 13, 8, 11, 15, and 12 *CBF* genes were identified from the genomes of *Xzs4, Xwf8, Xjg, Xg11*, and *Xzg2*, respectively. *CBF1-8* genes were present in all five genomes. *CBF9-CBF13* were dispensable genes present in genomes except *Xwf8*. Two unique cultivar-specific genes *CBF14* and *CBF15* were identified that were present only in the *Xg11* genome.

All the sequences contain the conserved AP2 domain. The physical and chemical properties of all CBF proteins were analyzed. There were no significant differences in amino acid residue number (AA), molecular weight (MW), isoelectric point (pI), instability index (II), aliphatic index (AI), and GRAVY among the five cultivars. The protein length ranged from 171-505 aa, MW ranged from 18.41-56.60 kDa, pI ranged from 4.61-9.70, II ranged from 43-73, AI ranged from 49.12-87.33, and GRAVY value ranged from -0.203 to -0.964. Most of the CBFs were localized in the nucleus (**Table 1**).

**Table 1:** Characteristics of 59 *CBF* genes in five yellowhorn cultivars.

Name	Gene	Chr	Start	End	Strand	AA	MW	Ip	II	AI	GRAVY	Subcellular Localization
*Xzs4_CBF1*	EVM0000338	Chr3	33884102	33884695	+	197	21797.67	4.609949	61.38325	61.92893	-0.56193	​Cytoplasm
*Xzs4_CBF2*	EVM0012620	Chr4	16342842	16343498	–	218	23888.79	5.487373	66.07523	70.73394	-0.28165	Nucleus
*Xzs4_CBF3*	EVM0005981	Chr11	25619969	25620652	–	227	25142.81	5.037265	59.7696	73.17181	-0.52775	Nucleus
*Xzs4_CBF4*	EVM0005738	Chr11	25623384	25624058	–	224	24934.69	5.116726	55.4683	71.96429	-0.48705	Nucleus
*Xzs4_CBF5*	EVM0002591	Chr11	25633381	25634664	–	205	22749.5	7.712903	59.06829	78.09756	-0.47463	Nucleus
*Xzs4_CBF6*	EVM0002707	Chr11	25646290	25652327	–	234	26220.24	5.495103	51.77949	73.11966	-0.50641	Nucleus
*Xzs4_CBF7*	EVM0017184	Chr11	25660715	25661398	–	227	25016.87	5.027489	45.62555	78.32599	-0.38899	Nucleus
*Xzs4_CBF8*	EVM0002147	Chr11	25675706	25676392	–	228	24710.24	5.147646	49.74474	66.84211	-0.62632	Mitochondria
*Xzs4_CBF9*	EVM0004174	Chr12	23889386	23890914	–	224	25153.27	6.245491	50.04464	62.90179	-0.49643	Nucleus
*Xzs4_CBF10*	EVM0001246	Chr12	23906031	23906546	–	171	18407.26	5.207327	61.58596	61.75439	-0.58304	Mitochondria
*Xzs4_CBF11*	EVM0016628	Chr13	27523812	27524471	+	219	24181.49	7.743199	71.98539	54.47489	-0.81963	Nucleus
*Xzs4_CBF12*	EVM0007008	Chr13	27539028	27540486	+	235	26400.48	9.704506	58.72553	58.25532	-0.79149	Nucleus
*Xzs4_CBF13*	EVM0017076	Chr13	27586709	27587323	–	204	21778.91	5.393645	56.3902	70.04902	-0.39461	Chloroplast
*Xwf8_CBF1*	XS01G00649	Chr1	6557308	6563676	–	505	56596.05	6.054569	51.46099	69.16832	-0.59624	Nucleus
*Xwf8_CBF2*	XS02G10797	Chr2	33824715	33825698	+	197	21811.7	4.609949	59.04518	61.92893	-0.56142	​Cytoplasm
*Xwf8_CBF3*	XS10G03018	Chr10	4927963	4929265	+	219	23384.77	5.138666	70.43881	61.55251	-0.61918	Nucleus
*Xwf8_CBF4*	XS10G03020	Chr10	4945033	4946017	+	237	26075.87	4.84117	52.68819	61.51899	-0.46751	Nucleus
*Xwf8_CBF5*	XS13G06172	Chr13	1326350	1327638	+	228	24710.24	5.147646	49.74474	66.84211	-0.62632	Mitochondria
*Xwf8_CBF6*	XS13G06174	Chr13	1351200	1352622	+	303	33773.14	8.268086	50.28581	87.32673	-0.27393	Chloroplast
*Xwf8_CBF7*	XS13G06177	Chr13	1377680	1378360	+	226	25035.84	5.332941	55.86327	74.29204	-0.4677	Nucleus
*Xwf8_CBF8*	XS13G06178	Chr13	1385326	1389570	+	227	25309.01	4.972127	60.29427	70.57269	-0.57225	Nucleus
*Xjg_CBF1*	XS07G0175500	Chr7	16676325	16676981	+	218	23888.79	5.487373	66.07523	70.73394	-0.28165	Nucleus
*Xjg_CBF2*	XS11G0182400	Chr11	22646316	22647029	–	237	26075.87	4.84117	52.68819	61.51899	-0.46751	Nucleus
*Xjg_CBF3*	XS11G0182700	Chr11	22661982	22662641	–	219	23384.77	5.138666	70.43881	61.55251	-0.61918	Nucleus
*Xjg_CBF4*	XS12G0008300	Chr12	766416	768911	+	314	35522.79	5.404104	68.11656	50.41401	-0.96369	Nucleus
*Xjg_CBF5*	XS12G0009400	Chr12	836129	836746	–	205	21851.96	5.393645	59.58439	69.70732	-0.39707	Chloroplast
*Xjg_CBF6*	XS13G0038500	Chr13	3422471	3423674	+	236	25783.72	6.438005	50.03559	66.65254	-0.56059	Mitochondria
*Xjg_CBF7*	XS13G0038700	Chr13	3438704	3439387	+	227	24959.82	5.205622	46.07797	76.60793	-0.41233	Nucleus
*Xjg_CBF8*	XS13G0039000	Chr13	3453599	3459713	+	200	22171.83	5.740761	61.87505	74.8	-0.4285	Nucleus
*Xjg_CBF9*	XS13G0039200	Chr13	3478296	3478976	+	226	25035.84	5.332941	55.86327	74.29204	-0.4677	Nucleus
*Xjg_CBF10*	XS13G0039300	Chr13	3488280	3488954	+	224	24934.69	5.116726	55.4683	71.96429	-0.48705	Nucleus
*Xjg_CBF11*	XS13G0039400	Chr13	3491642	3492325	+	227	25170.87	5.128264	57.74053	73.17181	-0.53216	Nucleus
*Xg11_CBF1*	Xso_Chr02_02907	Chr2	33907198	33907791	+	197	22264.22	4.931487	65.62462	57.38693	-0.64221	​Cytoplasm
*Xg11_CBF2*	Xso_Chr05_01501	Chr5	17498843	17499499	+	218	24341.31	6.819338	71.83318	66.54545	-0.35727	Nucleus
*Xg11_CBF3*	Xso_Chr10_00492	Chr10	6812419	6813078	+	219	23765.23	6.313812	71.92443	57.46606	-0.67738	Nucleus
*Xg11_CBF4*	Xso_Chr10_00493	Chr10	6828202	6828915	+	237	26528.39	5.110701	55.76946	57.74059	-0.53556	Nucleus
*Xg11_CBF5*	Xso_Chr13_00407	Chr13	3346447	3347133	+	228	25286.15	5.317026	49.24254	74.5614	-0.46447	Nucleus
*Xg11_CBF6*	Xso_Chr13_00408	Chr13	3361997	3362680	+	227	34117.71	8.675204	46.52724	82.98077	-0.20321	Nucleus
*Xg11_CBF7*	Xso_Chr13_00409	Chr13	3372979	3374580	+	309	30067.93	7.101373	53.87406	81.50376	-0.25301	Chloroplast
*Xg11_CBF8*	Xso_Chr13_00410	Chr13	3383274	3384065	+	263	25160.94	5.304806	56.544	69.91111	-0.52311	Nucleus
*Xg11_CBF9*	Xso_Chr13_00412	Chr13	3396403	3397083	+	226	25369.07	5.21483	60.81228	71.14035	-0.56316	Nucleus
*Xg11_CBF10*	Xso_Chr13_00414	Chr13	3404138	3404812	+	224	25337.21	5.740875	59.1022	70.52863	-0.52203	Nucleus
*Xg11_CBF11*	Xso_Chr13_00415	Chr13	3408292	3408975	+	227	24936.5	5.428715	50.82664	64.84716	-0.66114	Nucleus
*Xg11_CBF12*	Xso_Chr14_02233	Chr14	27536596	27537210	+	204	22217.4	5.890532	63.93204	65.58252	-0.47476	Mitochondria
*Xg11_CBF13*	Xso_Chr14_02238	Chr14	27565703	27566766	–	279	31885.36	8.291811	58.17107	65.57143	-0.71214	Nucleus
*Xg11_CBF14*	Xso_Chr14_02243	Chr14	27589481	27590146	–	221	24506.94	7.749508	70.19457	52.66968	-0.78145	Nucleus
*Xg11_CBF15*	Xso_Chr14_02247	Chr14	27604677	27605342	–	221	24712.2	8.413011	72.99865	53.73874	-0.80541	Nucleus
*Xzg2_CBF1*	Xsorbifolium007924.1	Chr5	2507676	2508787	–	197	21811.7	4.609949	59.04518	61.92893	-0.56142	​Cytoplasm
*Xzg2_CBF2*	Xsorbifolium011078.1	Chr6	16943261	16944430	+	218	23874.76	5.487373	66.95872	70.27523	-0.27982	Nucleus
*Xzg2_CBF3*	Xsorbifolium017345.1	Chr10	26777559	26778886	–	237	26119.88	4.790697	52.05401	61.09705	-0.48987	Nucleus
*Xzg2_CBF4*	Xsorbifolium017346.1	Chr10	26793571	26795196	–	171	18407.26	5.207327	61.58596	61.75439	-0.58304	Mitochondria
*Xzg2_CBF5*	Xsorbifolium018921.1	Chr12	766480	767209	+	221	24485.94	7.743199	71.98281	55.74661	-0.77014	Nucleus
*Xzg2_CBF6*	Xsorbifolium018924.1	Chr12	786421	787070	+	205	22648.73	6.20866	73.00244	49.12195	-0.86195	Nucleus
*Xzg2_CBF7*	Xsorbifolium018934.1	Chr12	848979	849593	–	204	21764.88	5.393645	58.88333	70.04902	-0.3951	Mitochondria
*Xzg2_CBF8*	Xsorbifolium020780.1	Chr13	3623971	3625226	+	228	24710.24	5.147646	49.74474	66.84211	-0.62632	Mitochondria
*Xzg2_CBF9*	Xsorbifolium020781.1	Chr13	3640044	3641082	+	227	24844.64	5.101436	43.42775	74.88987	-0.41806	Nucleus
*Xzg2_CBF10*	Xsorbifolium020782.1	Chr13	3649784	3650467	+	227	25252.03	5.324586	50.66872	76.1674	-0.47357	Nucleus
*Xzg2_CBF11*	Xsorbifolium020785.1	Chr13	3674063	3674959	+	226	25035.84	5.332941	55.86327	74.29204	-0.4677	Nucleus
*Xzg2_CBF12*	Xsorbifolium020786.1	Chr13	3683915	3688390	+	227	25309.01	4.972127	60.29427	70.57269	-0.57225	Nucleus

+ mean gene is present in farward strand and - mean gene is present in reverse strand.

### 3.2 Phylogenetic relationships of CBF family members from five yellowhorn cultivars

**Figure 1:**
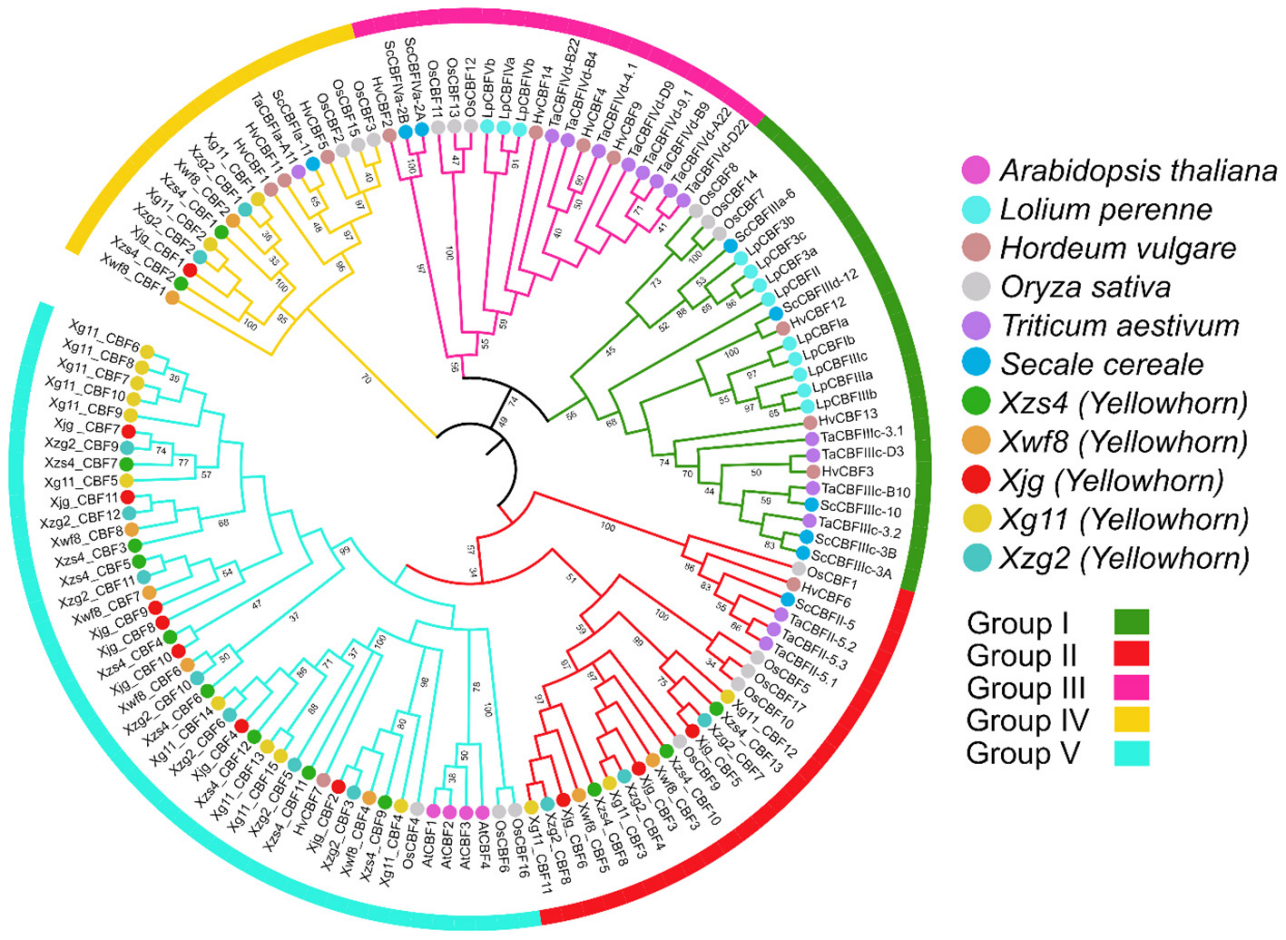
An ML phylogenetic tree (circular tree) constructed with full-length amino acid sequences of the 129 CBFs, 1000 bootstrap replicates, and set JTT+F+I+G4 best-fit substitution model through IQ Tree software. The tree was viewed in the iTOL software and divided into five groups: pink, yellow, green, cyan, and red.

To analyze the possible evolutionary characteristics of the CBF gene family in yellowhorn, we constructed a phylogenetic tree based on 129 CBF amino acid sequences including 4 from *A. thaliana*, 12 from *L. perenne*, 12 from *H. vulgare*, 17 from *O. sativa*, 16 from *T. aestivum*, 9 from *S. cereale*, and 59 from five yellowhorn cultivars. All CBF proteins were clustered into five groups. Group V contained the most *CBF* gene family members, followed by group I, II, III, and Group IV had the fewest members. Group I and II had the same number of *CBF* genes (**Figure 1**).

### 3.3 Exon-Intron and conserved motif distribution analysis of the CBF family

**Figure 2:**
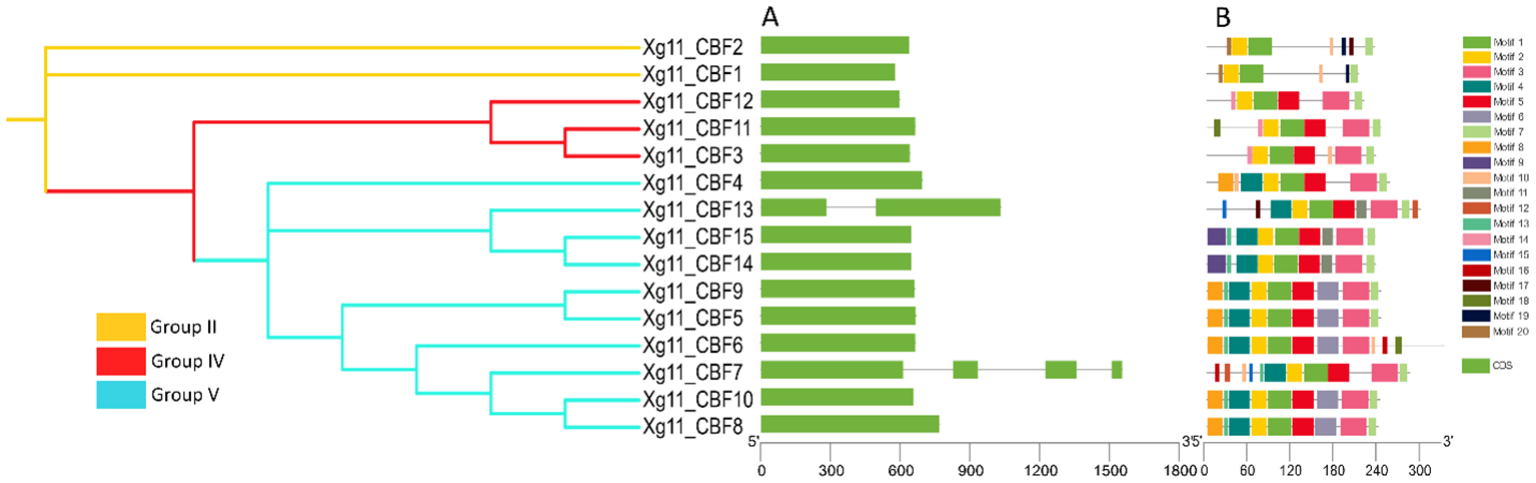
Structural and motif analyses of Xg11_CBFs. **(A)** Exon/intron structures of CBFs, **(B)** Schematic representation of the conserved motif compositions.

To gain insight into potential functions and diversification among CBFs, the exon–intron organizations and encoded conserved motifs were compared. As expected, most phylogenetically closely related STPs shared similar motifs and structures (**Figure 2**). The exon/intron structures exhibited a highly conserved organization in *CBF* genes. Most of the CBFs presented one exon while few members have more than one exon. Maximum number of exons observed for CBF2 was 4. Twenty predicted motifs were identified throughout the CBF protein sequences. Motifs 1 and 5 were present in all analyzed CBFs. Motifs 2, 3, and 4 were also present in most of the members. Motif 7 was only conserved among all the members of *Xg11* and motif 6 was only conserved among all the members of *Xzg2* cultivars (**Supplementary Figure S1**-**S4**).

### 3.4 Chromosomal mapping and duplication events

**Figure 3:**
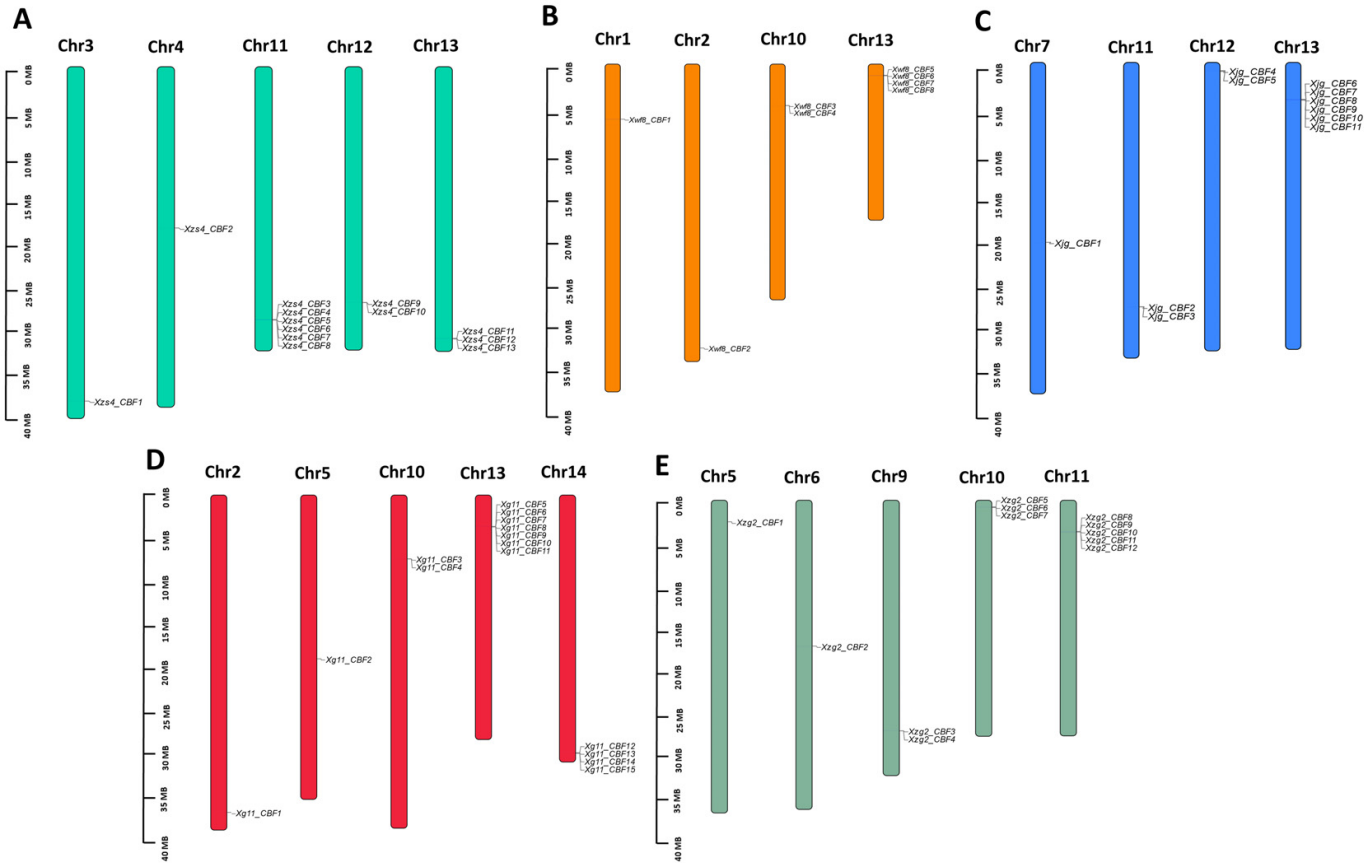
Localization of *CBF* genes on chromosomes. **(A)** *Xzs4_CBF* **(B)** *Xwf8_CBF*, **(C)** *Xjg_CBF*, **(D)** *Xg11_CBF*, and **(E)** *Xzg2_CBF*. Each chromosome representation displays the chromosomal number at the top.

All *CBF* genes of five yellowhorn cultivars were located on 15 chromosomes. The *CBFs* were mapped on their corresponding position on chromosomes. In *Xg11*, most of the *CBF* genes were located on Chr13 followed by Chr14. Chr 2, 5, and 10 contained 1,1, and 2 *CBF* genes, respectively (**Figure 3D**). The other four cultivars contained most of the genes on Chr13. In all five yellowhorn cultivars Chr8 and 15 contained no *CBF* genes localized on them (**Figures 3A–C, E**).

According to the defined criteria, the analysis of gene duplication events showed that there were 44 pairs of tandem duplication genes in the five yellowhorn cultivars including 11 duplicated pairs in *Xzs4*, 3 in *Xwf8*, 9 in *Xjg*, 14 in *Xg11*, and 7 in *Xzg2*. No segmental duplicated pair was observed in all of the five cultivars. The unique genes *Xg11_CBF13* and *Xg11_CBF14* were found to exhibit tandem duplication. The Ka/Ks ratio ranged from 0.24-1.93 and the duplication time was observed from 0.35-5.07 Mya (**Table 2**).

**Table 2:** Duplicated pairs of five yellowhorn genes, synonymous and non-synonymous mutations, duplication time, and type of duplication between the genes.

Gene 1	Gene 2	Ka	Ks	Ka/Ks	Time (MYA)	Duplication Type
*Xzs4_CBF3*	*Xzs4_CBF4*	0.0395	0.0541	0.73	1.80	Tandem
*Xzs4_CBF3*	*Xzs4_CBF5*	0.0464	0.0536	0.87	1.79	Tandem
*Xzs4_CBF3*	*Xzs4_CBF6*	0.0628	0.1152	0.55	3.84	Tandem
*Xzs4_CBF3*	*Xzs4_CBF7*	0.0294	0.0768	0.38	2.56	Tandem
*Xzs4_CBF4*	*Xzs4_CBF5*	0.0262	0.0421	0.62	1.40	Tandem
*Xzs4_CBF4*	*Xzs4_CBF6*	0.0656	0.0931	0.70	3.10	Tandem
*Xzs4_CBF4*	*Xzs4_CBF7*	0.0396	0.076	0.52	2.53	Tandem
*Xzs4_CBF5*	*Xzs4_CBF6*	0.077	0.126	0.61	4.20	Tandem
*Xzs4_CBF5*	*Xzs4_CBF7*	0.05	0.0753	0.66	2.51	Tandem
*Xzs4_CBF6*	*Xzs4_CBF7*	0.0662	0.1522	0.43	5.07	Tandem
*Xzs4_CBF11*	*Xzs4_CBF12*	0.0331	0.0204	1.62	0.68	Tandem
*Xwf8_CBF6*	*Xwf8_CBF7*	0.0417	0.0868	0.48	2.89	Tandem
*Xwf8_CBF6*	*Xwf8_CBF8*	0.0389	0.0973	0.40	3.24	Tandem
*Xwf8_CBF7*	*Xwf8_CBF8*	0.0497	0.0692	0.72	2.31	Tandem
*Xjg_CBF7*	*Xjg_CBF8*	0.0544	0.1124	0.48	3.75	Tandem
*Xjg_CBF7*	*Xjg_CBF9*	0.0486	0.0821	0.59	2.74	Tandem
*Xjg_CBF7*	*Xjg_CBF10*	0.0514	0.0731	0.70	2.44	Tandem
*Xjg_CBF7*	*Xjg_CBF11*	0.0253	0.0825	0.31	2.75	Tandem
*Xjg_CBF8*	*Xjg_CBF9*	0.0544	0.0825	0.66	2.75	Tandem
*Xjg_CBF8*	*Xjg_CBF10*	0.0455	0.0831	0.55	2.77	Tandem
*Xjg_CBF8*	*Xjg_CBF11*	0.0544	0.0732	0.74	2.44	Tandem
*Xjg_CBF9*	*Xjg_CBF10*	0.0397	0.0356	1.12	1.19	Tandem
*Xjg_CBF9*	*Xjg_CBF11*	0.0456	0.0446	1.02	1.49	Tandem
*Xg11_CBF6*	*Xg11_CBF7*	0.048	0.0332	1.45	1.11	Tandem
*Xg11_CBF6*	*Xg11_CBF8*	0.0615	0.0716	0.86	2.39	Tandem
*Xg11_CBF6*	*Xg11_CBF9*	0.0595	0.0421	1.41	1.40	Tandem
*Xg11_CBF6*	*Xg11_CBF10*	0.0639	0.0555	1.15	1.85	Tandem
*Xg11_CBF6*	*Xg11_CBF11*	0.0422	0.0332	1.27	1.11	Tandem
*Xg11_CBF8*	*Xg11_CBF9*	0.0583	0.0634	0.92	2.11	Tandem
*Xg11_CBF8*	*Xg11_CBF10*	0.0695	0.0385	1.81	1.28	Tandem
*Xg11_CBF8*	*Xg11_CBF11*	0.0644	0.0717	0.90	2.39	Tandem
*Xg11_CBF9*	*Xg11_CBF10*	0.0519	0.0298	1.74	0.99	Tandem
*Xg11_CBF9*	*Xg11_CBF11*	0.0536	0.0422	1.27	1.41	Tandem
*Xg11_CBF10*	*Xg11_CBF11*	0.055	0.0557	0.99	1.86	Tandem
*Xg11_CBF13*	*Xg11_CBF14*	0.0528	0.0273	1.93	0.91	Tandem
*Xg11_CBF13*	*Xg11_CBF15*	0.0383	0.0273	1.40	0.91	Tandem
*Xg11_CBF14*	*Xg11_CBF15*	0.0192	0.0168	1.14	0.56	Tandem
*Xzg2_CBF5*	*Xzg2_CBF6*	0.0162	0.0105	1.54	0.35	Tandem
*Xzg2_CBF9*	*Xzg2_CBF10*	0.0361	0.1502	0.24	5.01	Tandem
*Xzg2_CBF9*	*Xzg2_CBF11*	0.053	0.0891	0.59	2.97	Tandem
*Xzg2_CBF9*	*Xzg2_CBF12*	0.0393	0.0779	0.50	2.60	Tandem
*Xzg2_CBF10*	*Xzg2_CBF11*	0.0393	0.1014	0.39	3.38	Tandem
*Xzg2_CBF10*	*Xzg2_CBF12*	0.0426	0.0901	0.47	3.00	Tandem
*Xzg2_CBF11*	*Xzg2_CBF12*	0.046	0.0326	1.41	1.09	Tandem

### 3.5 PPI and GO enrichment analysis

**Figure 4:**
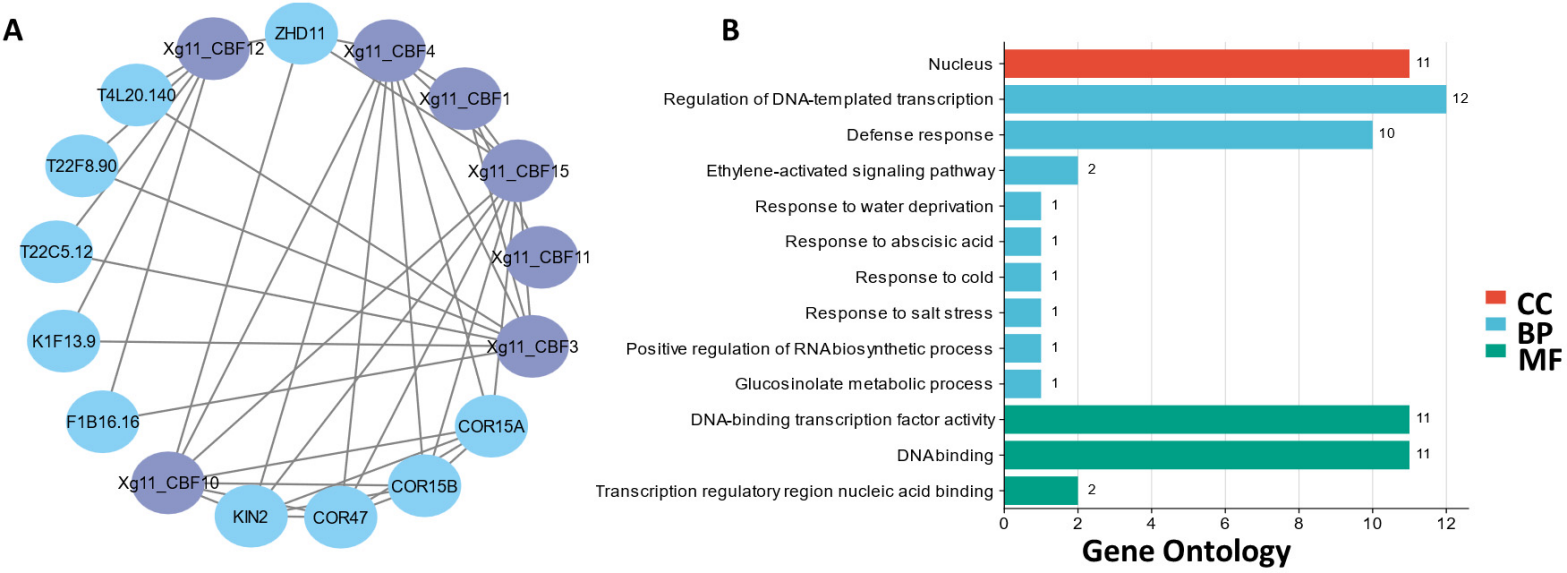
**(A)** Interactions among Xg11_CBF proteins and other homologous proteins. **(B)** Predicted biological processes (BP), cellular components (CC), and molecular functions (MF) associated with Xg11_*CBFs*.

A PPI network of Xg11_CBFs was generated to perform functional evaluation. The seven Xg11_CBFs including Xg11_CBF1, Xg11_CBF3, Xg11_CBF4, Xg11_CBF10, Xg11_CBF11, Xg11_CBF12, and Xg11_CBF15 showed interactions with other homologous proteins of *A. thaliana*. These homologous proteins were found to have higher expression in various abiotic stresses such as drought, salt, cold, and osmotic stresses, suggesting the potential roles of Xzs4_CBFs in abiotic stress regulation pathways (**Figure 4A**).

The GO enrichment analysis showed that these proteins are found in the nucleus component of the cell. Further, the Xg11_CBFs proteins are found to be involved in responses to water deprivation, cold, and salt stress. Moreover, these are also involved in the ethylene-activated signaling pathway and regulation of RNA biosynthetic pathway. Further, these are also involved in several MFs including DNA-binding transcription factor activity, and transcription regulatory region for nucleic acid binding (**Figure 4B**).

### 3.6 *Cis*-regulatory element analysis of yellowhorn *CBFs*

**Figure 5:**
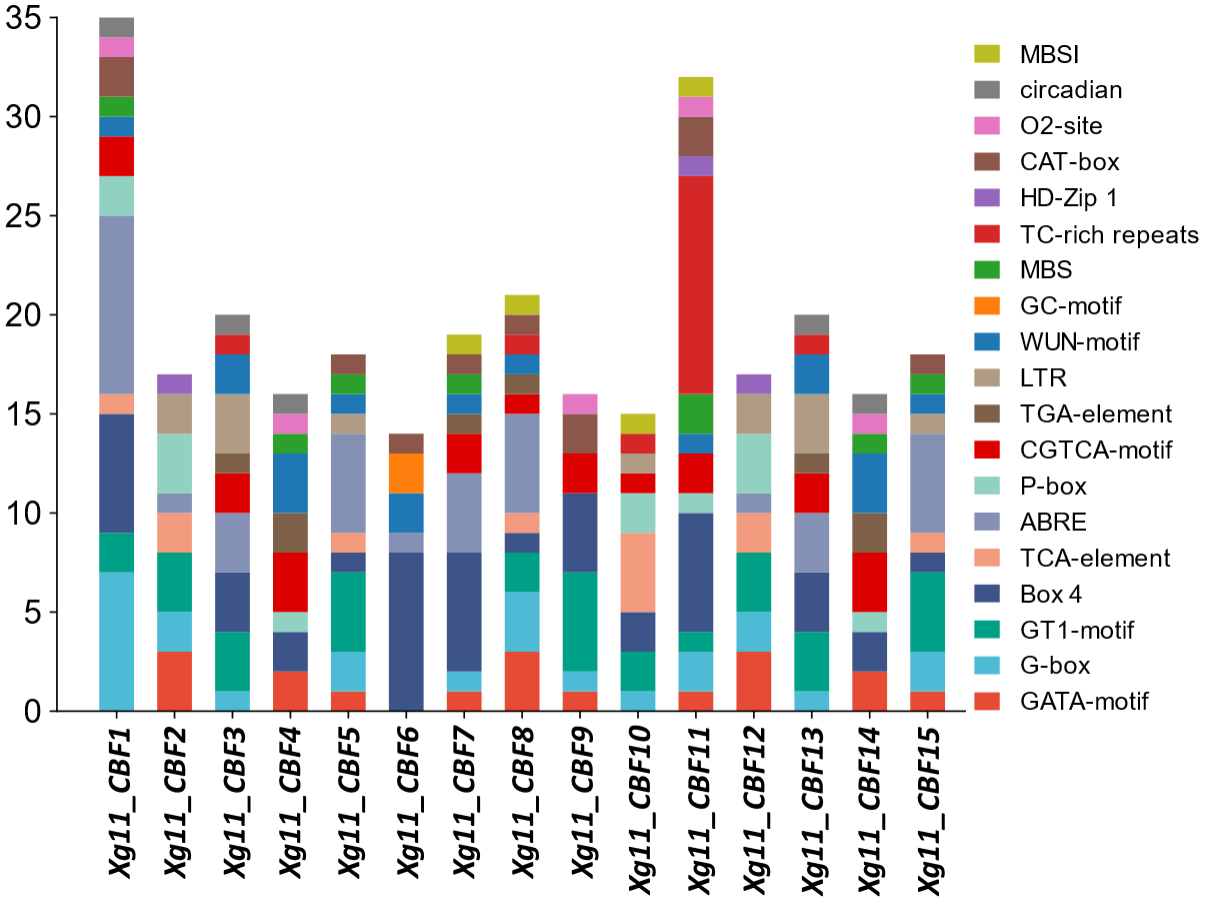
The *cis*-elements identified in the promoter regions of *Xg11_CBFs* genes.

To further evaluate the stress-responsive behaviors of yellowhorn *CBFs*, their promoter regions were analyzed to find stress-related *cis*-regulatory elements. The elements such as G-box, GATA-motif, Box 4, and GT1-motif were found in almost all identified members. These elements are associated with light stress regulation. Five elements including TGA-element, P-box, TCA-element, CGTCA-motif, and ABRE were found which are linked to hormone responsiveness. The stress-responsive elements; LTR, GC-motif, MBS, and TC-rich repeats were also found. Development-related elements which include CAT-box, MBSI, circadian, HD-Zip 1, and o2-site were found. All these elements show the potential involvement of yellowhorn *CBFs* genes in light stress, abiotic stresses, and development-related mechanisms (**Figure 5**, **Supplementary Figure S5**).

### 3.7 Expression profiling of Xg11 *CBFs* in tissues and abiotic stress

**Figure 6:**
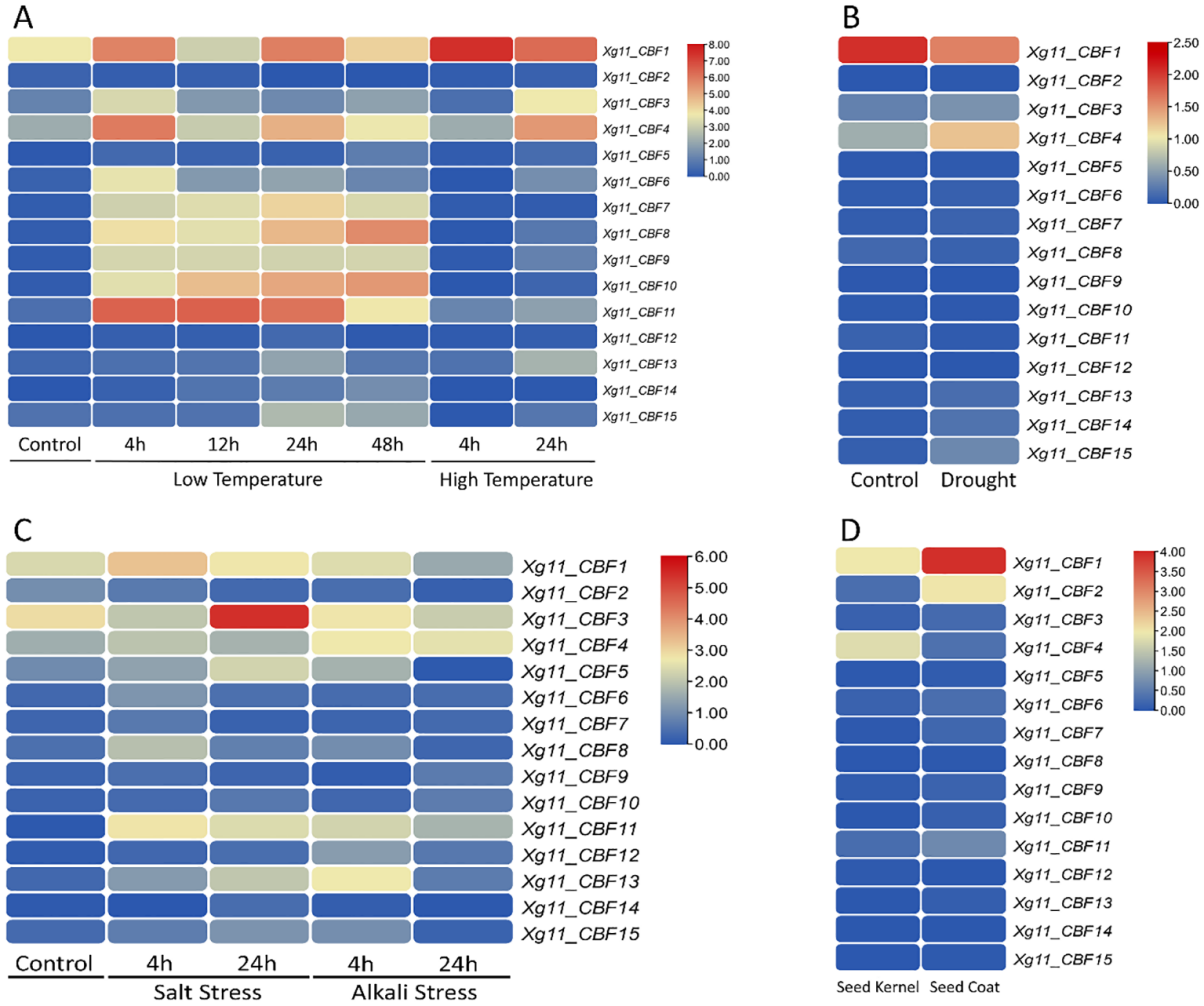
The expression profiles of Xg11 *CBF* genes in **(A)** low and high-temperature stress conditions, **(B)** drought stress, **(C)** salt, and alkali stress, and **(D)** in seed kernel and seed coat.

The expression profiling of Xg11 *CBF* genes was analyzed under low and high-temperature stress conditions (**Figure 6A**). Genes including *Xg11_CBF1*, *Xg11_CBF4*, *Xg11_CBF8*, *Xg11_CBF10*, and *Xg11_CBF11* showed change in expression levels under control, low-temperature, and high-temperature conditions. *Xg11_CBF11* was highly expressed during low-temperature stages and showed no change in expression under high-temperature. For drought stress, *Xg11_CBF1* showed a minimum change in expression and *Xg11_CBF4* contained an increase in expression (**Figure 6B**). Under salt and alkali stress conditions, *Xg11_CBF1* contained a fluctuated expression. *Xg11_CBF3* was highly expressed under salt treatment and contained a minimum change in expression in alkali conditions. Similarly, *Xg11_CBF11* also showed a change in expression pattern (**Figure 6C**). *Xg11_CBF1* was highly expressed in seed coat with minimal expression in seed kernel. *Xg11_CBF2* and *Xg11_CBF4* exhibited a change in expression pattern in seed kernel and coat (**Figure 6D**).

### 3.8 Phenotype, physical, and chemical characteristics of the transgenes under low-temperature stress

#### 3.8.1 Changes in growth phenotype of *A. thaliana* seedlings

The expression vector pEGOEP35S-H-*Xg11_CBF11* was transformed into *A. thaliana* plants with the floral-dip method. DNA was extracted and PCR amplified for eight resistant *A. thaliana* lines, of which 6 lines had a targeted band (**Supplementary Figure S6**). The expression levels of *Xg11_CBF11* in the transgenic plants were checked using qRT-PCR, the expression levels of transgenic L1, L5, L6 lines were higher than other lines (**Supplementary Table S2**), which were selected for the follow experiment.

**Figure 7:**
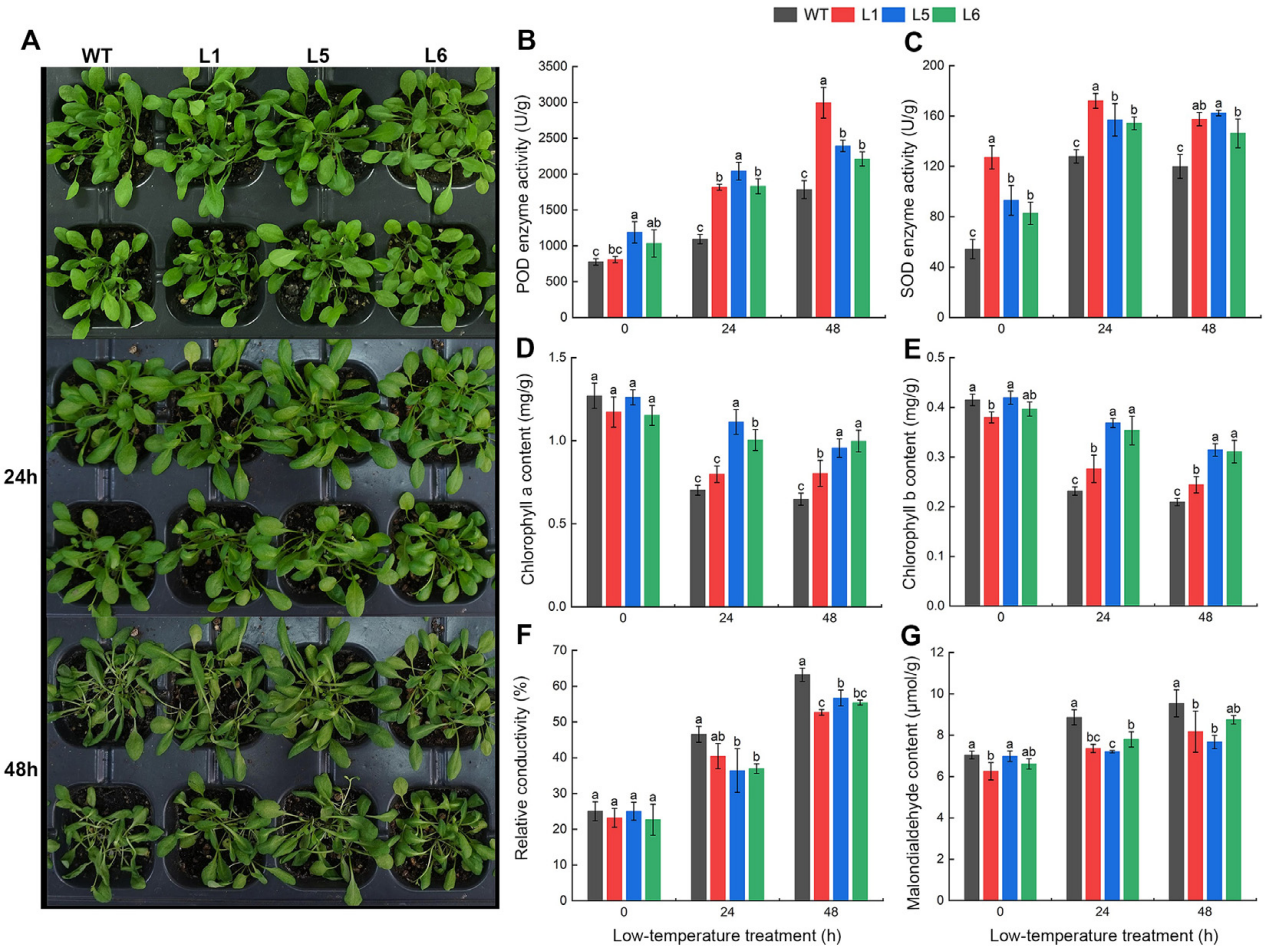
Growth phenotype and physiological indexes in wild-type (WT) and transgenic *A. thaliana* lines (L1, L5, L6) under 4°C cold stress at 0, 24, and 48h. **(A)** Growth phenotype. **(B)** POD enzyme activity. **(C)** SOD enzyme activity. **(D)** Chlorophyll a content. **(E)** Chlorophyll b content. **(F)** Relative conductivity. **(G)** Malondialdehyde (MDA) content. Different lowercase letters in the figure indicate significant differences (*p* < 0.05).

The growth of transgenic lines showed a non-significant difference compared to wild type before low-temperature stress. After low-temperature stress, all lines were affected to different degrees, and their leaves turned yellow and withered. At 24 h of cold stress, there was no significant change in the growth of each line. At 48 h of cold stress, the wilting degree of transgenic lines was light than that of wild type, indicating that transgenic *A. thaliana* lines were more cold-tolerance (**Figure 7A**).

#### 3.8.2 Changes in antioxidant enzyme activity of *A. thaliana* leaves

Continuous low-temperature stress increased the POD activity in all *A. thaliana* lines. The POD activity of the three transgenic lines were higher compared to wild type at every time point of low-temperature treatment. After 48 h of cold treatment, WT, L1, L5 and L6 increased by 1007.49 U/g, 2187.45 U/g, 1203.74 U/g and 1179.32 U/g, respectively, suggesting that transgenic lines had a greater increase than that of wild-type lines (**Figure 7B**). Transgenic lines showed significantly higher POD activity, indicating *Xg11_CBF11* enhanced antioxidant defense mechanisms compared to wild-type lines.

The SOD activity of L5 showed an upward trend while the other three lines increased first and then decreased. Meanwhile, the SOD activity of all transgenic lines was significantly higher than that of WT at any treatment time. At 0 h and 24 h of cold treatment, L1 had the highest SOD activity, which was 2.34 times and 1.34 times that of WT, respectively. At 48 h of cold treatment, L5 was the highest at 162.36 U/g, which was 1.35 times higher than that of WT (**Figure 7C**). Transgenic lines exhibited significantly higher SOD activity, further supporting enhanced antioxidant defenses compared to wild-type plants.

#### 3.8.3 Changes in chlorophyll contents of *A. thaliana* leaves

The chlorophyll a and chlorophyll b content decreased in all *A. thaliana* lines with increasing stress time, while the wild-type lines decreased more than the transgenic lines. Under normal culture conditions (0 h), there was no significant difference in chlorophyll indexes of the three transgenic lines compared with the wild-type lines. At 24 h and 48 h of cold treatment, the chlorophyll index of the wild-type lines was significantly lower than that of the transgenic lines (**Figures 7D, E**). Transgenic lines showed higher chlorophyll content compared to wild-type, indicating better preservation of photosynthetic pigments and maintenance of photosynthesis under stress.

#### 3.8.4 Changes in the membrane permeability of *A. thaliana* leaves

The relative conductivity of each *A. thaliana* line was constantly increased during the continuous low-temperature treatment. Under untreated conditions (0 h), the relative conductivity of seedling leaves was low, ranging from 20 to 30%, and the differences among different lines were not obvious. After 48 h of low-temperature stress, the relative conductivity of all lines exceeded 50%, while the wild-type lines were significantly higher than the transgenic lines (**Figure 7F**). Transgenic lines exhibited lower increases in relative conductivity, indicating reduced cell membrane damage compared to wild-type plants.

With the prolongation of cold stress time, the malondialdehyde (MDA) content in all *A. thaliana* lines increased continuously. After 48 h of low-temperature treatment, the MDA content of WT, L1, L5, and L6 lines increased by 35.6%, 30.7%, 9.8%, and 32.4%, respectively, compared to before low-temperature treatment (**Figure 7G**). Transgenic lines exhibited lower MDA content, suggesting reduced lipid peroxidation and cell membrane damage compared to wild-type plants.

## 4 Discussion

*CBF* gene families have been identified in several plant cultivars and sequences have also been analyzed in *A. thaliana* (Novillo et al., 2012), rice (*O. sativa)* (Cao et al., 2008), ryegrass *(L. perenne)* (D. Wang et al., 2023), rye (*S. cereal*) (Jung and Seo, 2018), wheat (*T. aestivum*) (Mohseni et al., 2012), cotton (*G. hirsutum*) (Ma et al., 2014), and barley (*H. Vulgare*) (Choi et al., 2002). In this study, we identified 13, 8, 11, 15, and 12 *CBF* genes from the five yellowhorn cultivars (*Xzs4, Xwf8, Xjg, Xg11*, and *Xzg2*). Only eight genes were present in all yellowhorn varieties. Five genes were dispensable genes present in different cultivars but not in all. Two unique genes were present in only one variety, *Xg11*. A similar phenomenon was observed in other cultivars also. For example, in a recent maize pan-genome-wide study, 20 out of 30 genes were present in all varieties (Sun et al., 2023). Similarly, the rice TPS gene family analysis showed that one gene was absent in the reference genome (Sun et al., 2022). Phylogenetic analysis revealed that among the cultivars under study, yellowhorn CBF sequences are more closely related to those of *T. aestivum*, *O. sativa*, and *H. vulgare* as compared to others. The protein length of all five yellowhorn cultivars is almost equal to the length of reported *CBF* genes in other plants. Most of the CBFs were localized in the nucleus and few localized in the cytoplasm, and mitochondria, while in *S. cereale* the CBFs were localized in the same subcellular localization. Furthermore, the phylogenetic tree revealed that all the yellowhorn CBFs were present in three groups. The same trend was observed in *L. perenne*. (D. Wang et al., 2023). The gene structure and motif patterns were almost conserved in all CBFs. Motif 7 was only conserved in the *Xg11* and motif 6 was only conserved in the *Xzg2* cultivars. The motif conservation pattern of all five yellowhorn cultivars is similar to the *L. perenne* (D. Wang et al., 2023) and *S. cereale* (Jung and Seo, 2018). CBFs were distributed unevenly on chromosomes for all five yellowhorn cultivars. Tandem duplication is the major factor behind the duplication of *CBF* genes and these are duplicated about 0.35-5.07 Mya ago. This trend of chromosomal distribution and duplication was also similar in *O. sativa* (Cao et al., 2008)*, S. cereal* (Jung and Seo, 2018), *T. aestivum* (Mohseni et al., 2012), *G. hirsutum* (Ma et al., 2014), and *H. Vulgare* (Choi et al., 2002). The *cis*-regulatory elements in the promoter region, the PPI, and GO insisted on the potential involvement of these *CBF* genes in abiotic stress responsiveness. Previously breakthroughs have been made in the mechanism of cold tolerance in plants, among which ICE-*CBF*-COR signaling cascade pathway is one of the main cold tolerance pathways in higher plants and plays a crucial role under cold stress (Feng et al., 2020). The expression profiling of these genes in low and high temperature; drought; salt and alkali; and in seed kernel and coat showed a similar expression pattern of these genes as shown in previous studies. It has been reported that *AtCBF2* negatively regulated *AtCBF3* and *AtCBF1*, while *AtCBF4* functioned in drought stress tolerance (Haake et al., 2002). Similarly, *AcerApseCBFs*, *AcyanCBF2*/4, *AtruCBF5* genes are involved in drought inducibility (Zhao et al., 2023). Moreover, the core genes present in all yellowhorn varieties were showing a change in expression pattern, and potential involvement of these genes in stress responsiveness. While, for dispensable genes, a higher expression of *Xg11_CBF7-11* was observed which showed these genes are potentially involved in temperature stress responsiveness in *Xg11* variety.

In nature, plants often encounter various abiotic stresses, among which low-temperature is a very important environmental stress factor, which is an important obstacle in agricultural and forestry production in China. Compared with animals, plants themselves cannot move freely, so a system of antioxidant protective enzymes has evolved to reduce stress damage. POD is one of the key enzymes of the enzymatic defense system in plants under stress conditions, and the enzyme activity rises when encountering stress. The study found that the POD enzyme activity was significantly higher than that of nontransgenic tobacco, which improved the cold resistance of tobacco (Gu et al., 2021). In this study, the POD enzyme activity in the transgenic *A. thaliana* lines was continuously increased under cold stress, and the increase was significantly higher than that in the wild-type lines. However, the change trend of SOD enzyme is complex and does not always rise under stress. The SOD enzyme activity may decline continuously, fall first and then rise, or remain unchanged. In this study, the SOD enzyme activity in the L5 lines rose linearly with the duration of low-temperature stress, while the WT, L1, and L6 lines increased first and then decreased. Low-temperature stress affects the normal growth and development of plants, reducing the effective photosynthetic area, resulting in blocked chlorophyll synthesis and reduced content (Song et al., 2021). After silencing *CBF1* in cucumber, the chlorophyll content in gene-silenced plants decreased more than in wild-type plants, indicating that the *CBF1* gene plays an important role in cold stress (Gupta et al., 2012). In this study, the chlorophyll content of both transgenic and wild-type *A. thaliana* lines was gradually decreased under continuous low-temperature stress, indicating that low-temperature inhibited the normal photosynthesis of *A. thaliana*; at 24 h and 48 h, the chlorophyll content of the transgenic lines was higher than that of the wild-type line, with a smaller reduction. Therefore, it can be speculated that the *Xg11_CBF11* gene has a protective effect on chlorophyll to ensure the chlorophyll content.

Low-temperature stress will cause peroxidation of cell membrane lipids, increase membrane permeability, and lead to an increase in relative conductivity and malondialdehyde content. Therefore, the degree of cell membrane damage is usually measured by the relative conductivity and malondialdehyde content (Dong et al., 2020). The species-specific *CBF* genes in yellowhorn cultivars may contribute to physiological responses such as improved antioxidant enzyme activity, reduced membrane damage, and higher chlorophyll retention under low temperatures. The phenotypic differences observed among the five yellowhorn cultivars may enhance the cold tolerance through species-specific motifs and expression profiles. Thus, the species-specific genes not only contribute to cold tolerance but may also play a role in defining the unique adaptive traits of each cultivar. The overexpression of *A. thaliana CBF1* gene in the transgenic lines changed the relative conductivity and malondialdehyde content less than that of the non-transgenic control verifying the cold resistance function of the *A. thaliana CBF1* gene (Akhtar et al., 2012). Similarly, the *A. thaliana CBF1* gene was transformed into grape and tobacco (Park et al., 2001; Siddiqua and Nassuth, 2011), and the relative conductivity of the transgenic lines was also lower than the non-transgenic lines after cold stress, which announced the cold resistance of grape and tobacco. Similar results were obtained in this study, and the relative conductivity and malondialdehyde in transgenic lines were less elevated than those in wild-type lines, indicating that the *Xg11_CBF11* gene protected the cell membrane of *A. thaliana* and reduced the degree of damage.

## 5 Conclusion

This study has identified and characterized the *CBF* gene family across five yellowhorn cultivars, providing new insights into their structural and functional diversity. Phylogenetic and motif analyses highlighted both conserved features and species-specific adaptations among these cultivars, with gene duplication, especially tandem duplications, playing a significant role in the expansion of this gene family. Expression profiling revealed the involvement of these genes in abiotic stress responses, particularly under cold conditions. Functional validation of the *Xg11_CBF11* gene in transgenic *A. thaliana* demonstrated its positive role in enhancing cold tolerance, as evidenced by increased antioxidant enzyme activity, maintained chlorophyll levels, and reduced cellular damage. These findings enrich our understanding of the molecular mechanisms behind cold stress tolerance in yellowhorn and provide potential targets for further genetic improvement in this species. This research contributes to the broader effort of improving plant resilience to environmental stress, with practical implications for agricultural and forestry applications.

## Data Availability

The original contributions presented in the study are included in the article/**Supplementary Material**. Further inquiries can be directed to the corresponding authors.
