# Supplementary material for: Comparative genomic profiling of CBFs pan-gene family in five yellowhorn cultivars and functional identification of Xg11_CBF11
Source: Front Plant Sci. 2024 Nov 19;15:1481358. doi: 10.3389/fpls.2024.1481358 (PMC11613637; doi:10.3389/fpls.2024.1481358)
Supplement: Supplementary file 1 [file DataSheet1.docx]

**
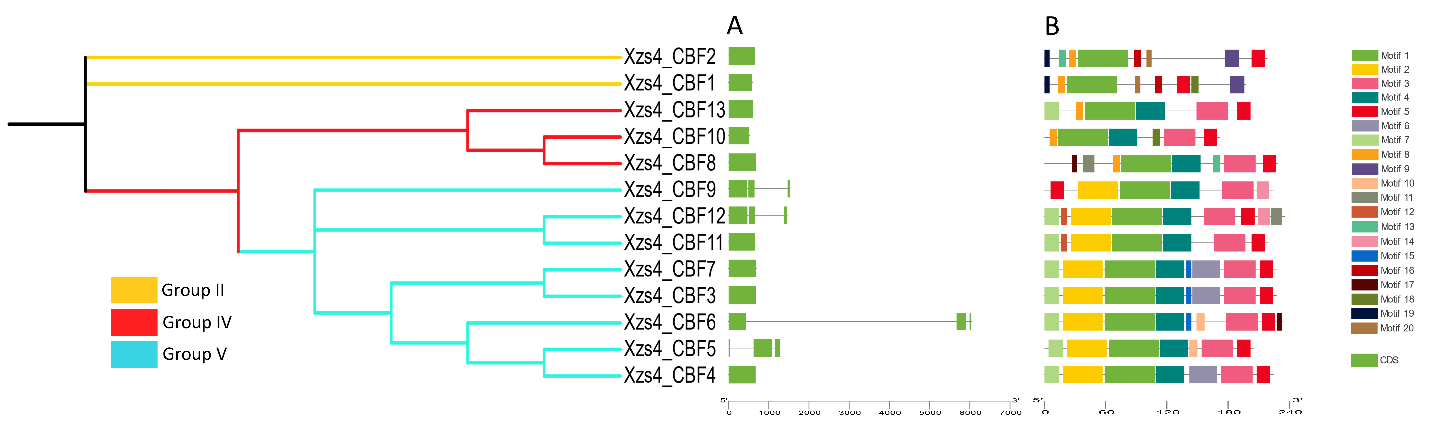
**

**Figure S1:** Structural and motif analyses of *Xzs4* AP2/CBFs. **(A)** Exon/intron structures of AP2/CBFs, **(B)** Schematic representation of the conserved motif compositions.


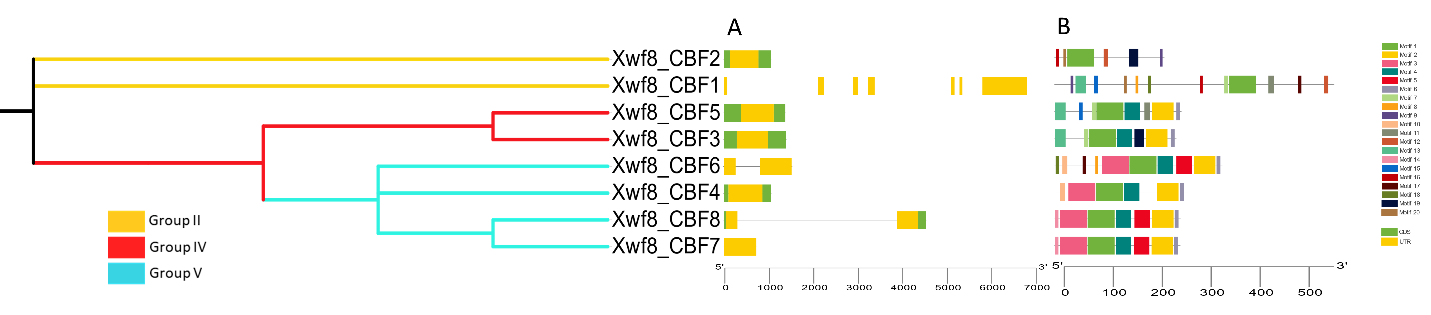


**Figure S2:** Structural and motif analyses of *Xwf8* AP2/CBFs. **(A)** Exon/intron structures of AP2/CBFs, **(B)** Schematic representation of the conserved motif compositions.


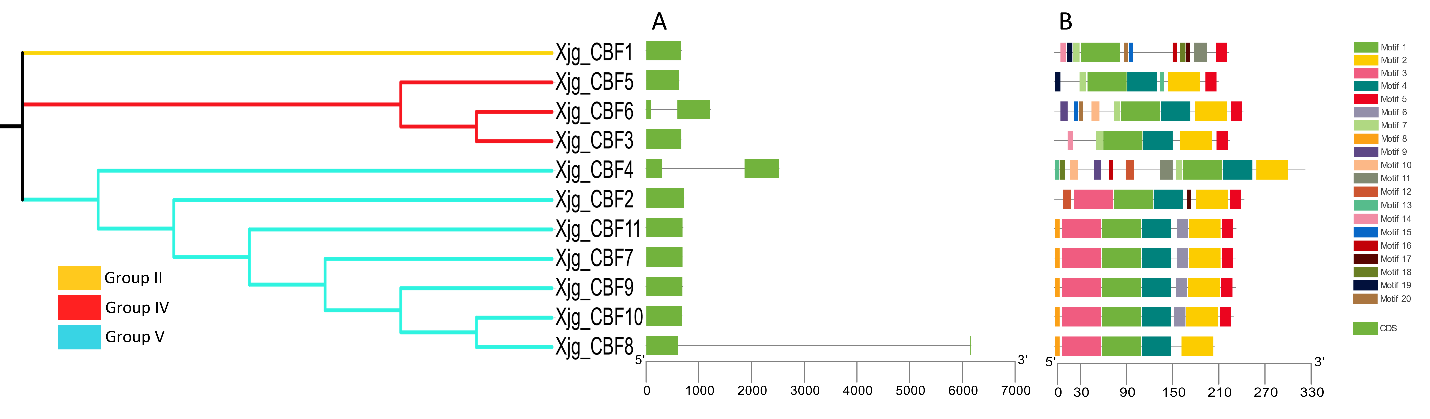


**Figure S3:** Structural and motif analyses of *Xjg* AP2/CBFs. **(A)** Exon/intron structures of AP2/CBFs, **(B)** Schematic representation of the conserved motif compositions.


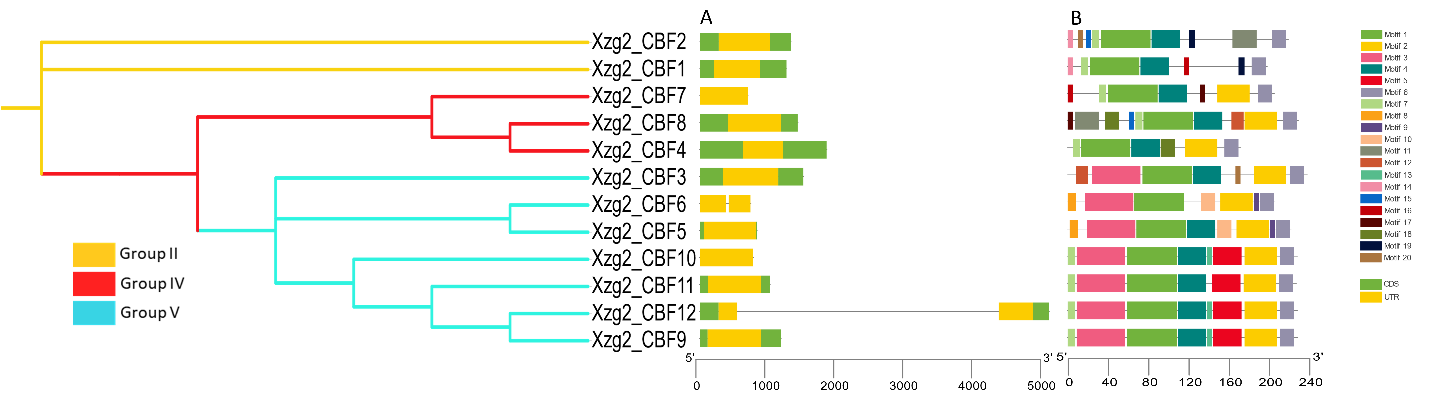


**Figure S4:** Structural and motif analyses of *Xzg2* CBFs. **(A)** Exon/intron structures of CBFs, **(B)** Schematic representation of the conserved motif compositions.


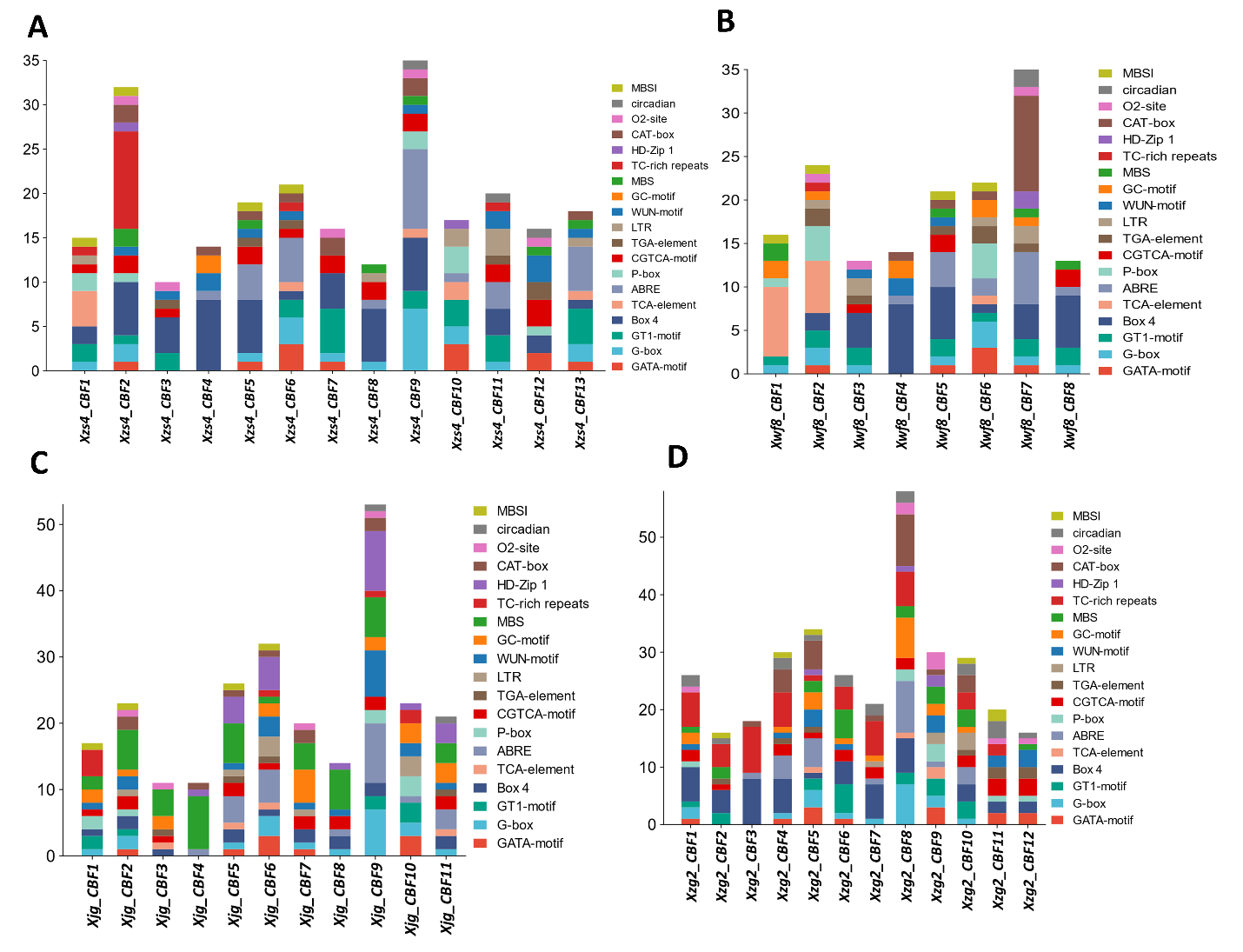


**Figure S5:** Cis-elements present in the promoter regions of the identified yellowhorn CBF genes.


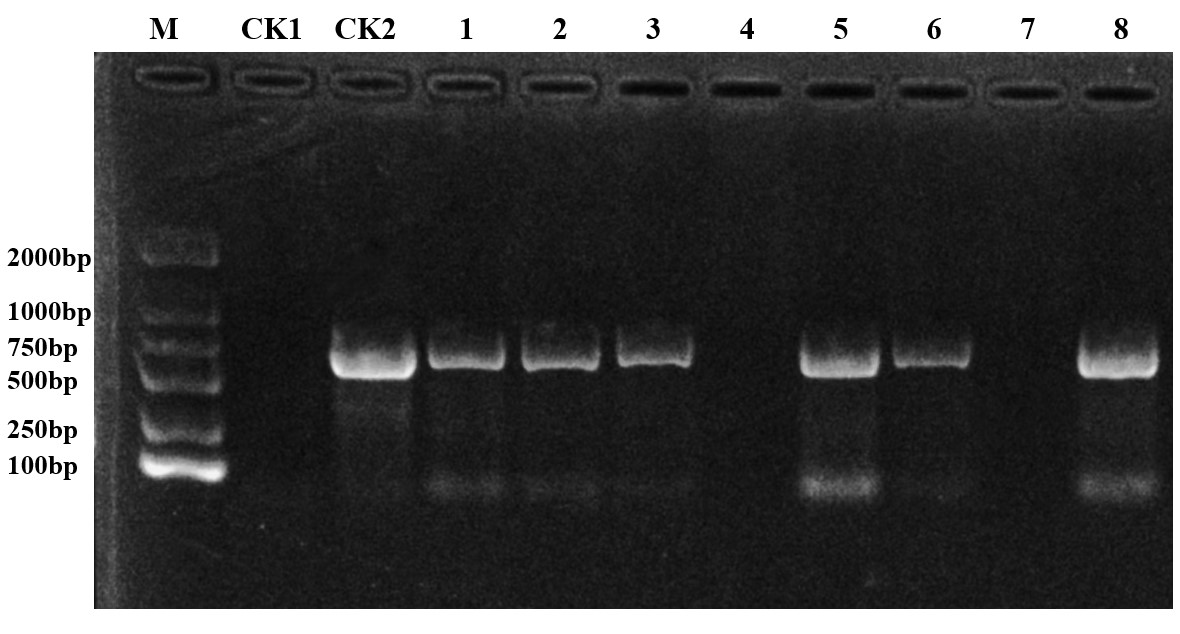


**Figure S6:** PCR identification of transgenic *A. thaliana* lines. M: DL2000 DNA Marker; CK1: wild-type control; CK2: positive plasmid; 1-8: transgenic *A. thaliana.*

**Table S1:** Primer sequences.

| **Primer name** | **Sequence 5’ to 3’** |
| --- | --- |
| *Xg11_CBF11*-F | ATGAACATCTTCAAGTCAGATAGTC |
| *Xg11_CBF11*-R | TCAAATTGAAAAACTCCAGAGTGG |
| *Xg11_CBF11*-e-F | ACTAGGGTCTCGCACCATGAACATCTTCAAGTCAGATAGTCCAGA |
| *Xg11_CBF11*-e-F | ACTAGGGTCTCTACCGTCAAATTGAAAAACTCCAGAGTGGAACAC |
| *Xg11_CBF11*-q-F | CTCGGATGAGGAGGTTCTG |
| *Xg11_CBF11*-q-R | CGCCTAACGCCTCTGTAT |
| *AtActin2*-F | GGTATCGCTGACCGTATGAG |
| *AtActin2*-R | GCTGAGGGAAGCAAGAATG |

**Table S2:** The expression analysis of *Xg11_CBF11* gene in the transgenic and wild-type *A. thaliana* lines*.*

| ***Xg11_CBF11*** | **Expression level** | **Average** | **Standard deviation** |
| --- | --- | --- | --- |
| WT | 1.086321304 | 1.048454425 | 0.374768 |
|  | 1.402851263 |  |  |
|  | 0.656190707 |  |  |
| LI | 7.937856734 | 7.66925021 | 0.764823 |
|  | 6.806357949 |  |  |
|  | 8.263535947 |  |  |
| L2 | 4.688978352 | 4.267712233 | 0.997959 |
|  | 3.12819666 |  |  |
|  | 4.985961687 |  |  |
| L3 | 4.914704368 | 5.106240881 | 0.762301 |
|  | 4.45797426 |  |  |
|  | 5.946044014 |  |  |
| L4 | 2.545958595 | 2.754327248 | 0.755169 |
|  | 3.59180393 |  |  |
|  | 2.125219219 |  |  |
| L5 | 9.88267102 | 10.71121399 | 1.220032 |
|  | 12.11220466 |  |  |
|  | 10.1387663 |  |  |
| L6 | 8.027440438 | 8.898274509 | 0.890649 |
|  | 8.859885462 |  |  |
|  | 9.807497628 |  |  |
